# Circulating Extracellular Vesicles Containing Xenobiotic Metabolizing CYP Enzymes and Their Potential Roles in Extrahepatic Cells Via Cell–Cell Interactions

**DOI:** 10.3390/ijms20246178

**Published:** 2019-12-07

**Authors:** Kelli Gerth, Sunitha Kodidela, Madeline Mahon, Sanjana Haque, Neha Verma, Santosh Kumar

**Affiliations:** Department of Pharmaceutical sciences, University of Tennessee Health Science Center, 881 Madison Avenue, Memphis, TN 38163, USA; kgerth1@uthsc.edu (K.G.); skodidel@uthsc.edu (S.K.); mmahon1@uthsc.edu (M.M.); shanque8@uthsc.edu (S.H.); neha.verma@smail.astate.edu (N.V.)

**Keywords:** extracellular vesicles, exosomes, cytochrome P450, extrahepatic tissues, plasma, circulatory CYPs

## Abstract

The cytochrome P450 (CYP) family of enzymes is known to metabolize the majority of xenobiotics. Hepatocytes, powerhouses of CYP enzymes, are where most drugs are metabolized into non-toxic metabolites. Additional tissues/cells such as gut, kidneys, lungs, blood, and brain cells express selective CYP enzymes. Extrahepatic CYP enzymes, especially in kidneys, also metabolize drugs into excretable forms. However, extrahepatic cells express a much lower level of CYPs than hepatocytes. It is possible that the liver secretes CYP enzymes, which circulate via plasma and are eventually delivered to extrahepatic cells (e.g., brain cells). CYP circulation likely occurs via extracellular vesicles (EVs), which carry important biomolecules for delivery to distant cells. Recent studies have revealed an abundance of several CYPs in plasma EVs and other cell-derived EVs, and have demonstrated the role of CYP-containing EVs in xenobiotic-induced toxicity via cell–cell interactions. Thus, it is important to study the mechanism for packaging CYP into EVs, their circulation via plasma, and their role in extrahepatic cells. Future studies could help to find novel EV biomarkers and help to utilize EVs in novel interventions via CYP-containing EV drug delivery. This review mainly covers the abundance of CYPs in plasma EVs and EVs derived from CYP-expressing cells, as well as the potential role of EV CYPs in cell–cell communication and their application with respect to novel biomarkers and therapeutic interventions.

## 1. Introduction

The cytochrome P450 (CYP) superfamily is a group of Phase I mono-oxidase enzymes with broad substrate specificity that is responsible for the majority of xenobiotic metabolism [1]. CYP enzymes are also involved in vital endogenous pathways, including prostaglandin metabolism and steroid hormone biosynthesis [2]. Gene names are determined according to a standardized nomenclature. Using CYP3A4 as an example, “CYP” refers to the superfamily of cytochrome P450 genes, “3” refers to the family designation (<40% amino acid identity with other CYPs), “A” refers to the subfamily designation (40–55% amino acid identity with other CYPs in that family), and “4” refers to the specific gene within the subfamily with >55% sequence identity [3]. This enzyme superfamily is ubiquitous in nature—it is present in bacteria, fungi, plants, and animals—with varying expression patterns [4]. In fact, many drug metabolizing isoforms including CYP 1A, 2C, and 3A show significant interspecies differences in enzymatic activity while CYP 2E1 does not [5].

Hepatocytes express an abundance of drug metabolizing CYP enzymes and demonstrate the greatest capacity for Phase I xenobiotic biotransformation, followed by the small intestine and kidneys [6,7,8]. Of the 57 known human CYP enzymes, five CYP isoenzymes from CYP 1–3 families metabolize the majority of clinically used drugs—CYP3A4/5, CYP2D6, CYP2C9, CYP2C19, and CYP1A2 [9]. Although CYP 1–3 families predominantly aid in xenobiotic detoxification, CYP enzymes are also involved in the bioactivation of xenobiotics, resulting in the formation of toxic intermediates. CYPs 1B1, 1A1, and 2A6 are involved in the bioactivation pathways of cigarette smoke constituents [10,11], while CYP2E1 is involved in the metabolism of alcohol and acetaminophen [12]. These are associated with the generation of hepatotoxic or carcinogenic metabolites and promote reactive oxygen species (ROS) production in vitro and in vivo [13,14,15], which eventually causes organ damage and cancers.

CYP enzymes are generally upregulated by their own substrates/drugs, resulting in enhanced metabolism and suboptimal plasma concentrations of concurrent drugs [16]. Conversely, CYP inhibition by various drugs contributes to supratherapeutic drug levels and drug-induced toxicity, thus preventing CYPs from performing their protective role in detoxification [16]. In the case of prodrugs that require CYP-mediated conversion to their active form, the reverse is true. Prototypical pharmacologic CYP enzyme inducers and inhibitors that are commonly implicated in drug–drug interactions include rifampin (inducer) and azole antifungals (inhibitors), which may interact with multiple CYP isoenzymes to varying degrees [9]. Further, pharmacogenetic variations in CYP activity may result in reduced, absent or increased metabolic capacity. Drug metabolizing isoforms with functionally relevant polymorphisms include CYPs 1A2, 2B6, 2C8, 2C9, 2C19, 2D6, and 3A4/5 [17,18]. Certain isoforms are more highly polymorphic than others [17] and are associated with clinically significant effects, such as toxicity or lack of therapeutic response [17]. Furthermore, while CYP enzymes are most abundantly expressed in the liver, they are also found in extrahepatic tissues throughout the body. Although the majority of extrahepatic CYPs are involved in endogenous pathways [2], drug metabolism also occurs outside the liver. The small intestine and kidneys are the primary sites of extrahepatic drug metabolizing CYP enzymes [6,7]; however, drug metabolizing CYPs are also expressed in the lungs, blood (monocytes, lymphocytes), brain, and heart [19,20,21,22]. Extrahepatic CYP enzymes contribute to cell-specific biotransformation, albeit to a lesser extent than hepatic CYPs. While extrahepatic CYP expression and metabolic capacity are not able to mediate total body clearance of xenobiotics, the enzymes may play a significant role in local tissue exposure and toxicity [19].

Recently, we provided the first evidence that functional CYP isoforms are packaged into extracellular vesicles (EVs) derived from human plasma of healthy volunteers, as well as in EVs derived from hepatic cell lines [23]. Extracellular vesicles (EVs) are nanosized, membrane-bound particles that are secreted from most cell types into biological fluids, namely plasma, and are taken up by other cells [24]. EV cargo includes a heterogeneous array of biomolecules, e.g., lipids, carbohydrates, cytokines, proteins, and nucleic acids—mRNAs, miRNAs, etc. [24,25,26]. Thus, EVs are thought to be critical in cell-to-cell signaling, protein transfer, and nucleic acid shuttling [24,27,28]. These characteristics suggest that EVs might be potential biomarkers, therapeutic targets, and drug-delivery systems [24].

It is important to note that exosomes are a subgroup of extracellular vesicles with a distinct biogenesis pathway [29]. Although much of the literature prior to 2018 refers to “exosomes”, distinguishing exosomes from other EVs has proved challenging due to overlaps in size, composition, and marker proteins [30]. Therefore, in accordance with current ISEV guidelines [30], this review will exclusively refer to “EVs” even when published reports refer to “exosomes”.

**Figure 1:**
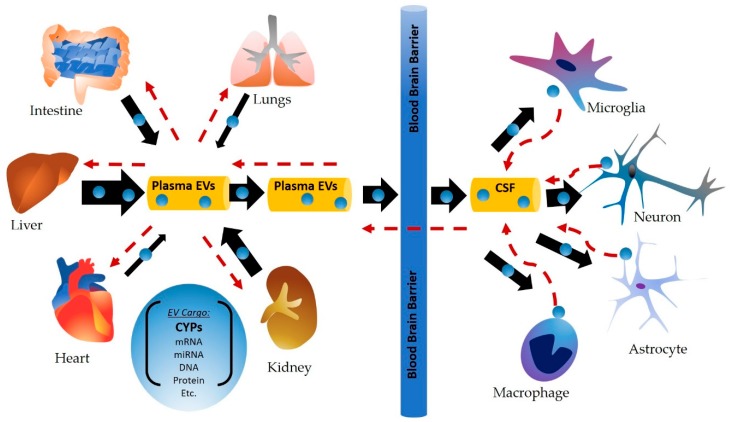
Cytochrome P450 (CYP)-containing plasma extracellular vesicles (EVs)/exosomes are secreted from the liver and other peripheral organs, circulate via plasma, and are delivered to distant sites (e.g., brain cells), where they may aid in extrahepatic drug metabolism, detoxification, and may also influence toxicity at these sites. Similarly, secretion of EVs from extrahepatic cells, including brain cells are also likely to contain CYPs in addition to other biomolecules, which would also be circulated via plasma and delivered to other distant cells.

As drug metabolic capacity is limited in extrahepatic tissues, it is possible that CYP-containing plasma EVs are secreted from the liver, circulate via plasma, and are delivered to distant sites (e.g., brain cells), where they may aid in extrahepatic drug metabolism, detoxification, and may also influence toxicity at these sites (Figure 1). It is also possible that extrahepatic cells in the kidneys, lungs, blood, heart, and brain also secrete EVs that are pooled in the plasma and cerebral spinal fluid (CSF), making an “EV-depot”. These EVs may then be delivered to other cells as needed and under specific conditions, to perform biological functions. This review will provide an overview of the contribution of CYPs to drug metabolism in extrahepatic tissues. Since our primary goal is to discuss relevant drug-metabolizing CYP enzymes and their xenobiotic substrates, discussion pertaining to endogenous pathways is largely outside the scope of this review. Importantly, we will summarize the most recent literature pertaining to CYPs and EVs, the relative abundance of CYPs in human plasma-derived EVs, and potential implications of CYP-containing EVs in xenobiotic biotransformation/bioactivation. Further, we will discuss the potential role of EV CYP enzymes as biomarkers in various pathological conditions and xenobiotic exposure/drug use, as well as suggest novel therapeutic interventions.

## 2. Expression of CYP Enzymes in Extrahepatic Tissues

Although the metabolic capacity of extrahepatic CYPs is relatively low compared to the capacity of hepatic CYPs, extrahepatic CYPs may still influence local tissue function and drug exposure, as well as drug biotransformation and bioactivation at these sites. Further, extrahepatic CYP enzymes may alter overall systemic exposure to xenobiotics, with corresponding elevations in toxicity [19,31]. Interestingly, some CYPs are expressed preferentially in extrahepatic tissues, which may lead to unique extrahepatic metabolites and tissue-specific consequences in cellular toxicity and organ pathology. The CYPs expressed in extrahepatic tissues are shown in Table 1.

### 2.1. Small Intestine

CYP3A4 is the main CYP isoform in the small intestine, accounting for roughly 82% of CYP enzymes expressed in gut tissue [8]. Many drugs, which are 3A4 substrates, have low oral bioavailability due in part to CYP-mediated intestinal first pass metabolism [8]. Due to CYP3A4’s broad substrate specificity and high expression in gut tissue, certain foods and dietary supplements can cause significant drug interactions. Grapefruit juice, a potent inhibitor of intestinal 3A4, is known to increase the plasma concentration of common 3A4 substrates, e.g., statins, calcium channel blockers, protease inhibitors, and many others [54]. Inhibition by grapefruit juice does not affect hepatic 3A4 but may decrease intestinal 3A4 function by 62% [32].

### 2.2. Kidneys

It has been estimated that the human kidney contains anywhere from 4–20% of hepatic CYP protein content [8]. Only CYPs 2B6 and 3A5 have been confirmed in the human kidney, and evidence for CYPs 3A4, 2C9, and 2C8 is equivocal [6]. Of these, CYP3A5 is the most prevalent isoform [19]. Renal CYP3A5 is highly polymorphic [19]. In fact, genetic variations in renal 3A5 expression may influence nephrotoxicity associated with the immunosuppressive agent, tacrolimus [55], as well as with the anticancer agent, ifosfamide [56]. Further, CYP3A forms are expressed consistently in renal cancer cells and may be involved in renal cancer development and multidrug resistance [57]. Nevertheless, renal CYP3A enzymes may also help suppress cancer via bioactivation of certain agents, forming metabolites that are cytotoxic to tumor cells and benign in noncancer cells [57].

### 2.3. Lungs

Lung tissue expresses CYPs 1A1, 1B1, 2A6, 2E1, 3A4, and 3A5 among others [8]. Many respiratory tract CYPs are linked to bioactivation of the constituents of cigarette smoke and enhanced toxicity and carcinogenicity. CYP1A1 is mostly expressed in smokers [8]. Both CYPs 1A1 and 1B1 isoforms are induced by compounds found in tobacco smoke, particularly Benzo(a)pyrene (Bap) [10,58]. Bap is a polycyclic aromatic hydrocarbon (PAH) carcinogen that is converted to DNA-reactive intermediates in a process dependent on CYP1A1 and CYP1B1 metabolic pathways [10]. CYP2A6 is mostly expressed in the trachea and is also thought to be involved in bioactivation of carcinogens from tobacco smoke [8]. CYP2A6 mainly metabolizes nicotine, the primary constituent in cigarette smoke, into cotinine and nicotine-derived nitrosamine ketone (NNK) [59]. Of note, CYP2A6 polymorphisms are involved in the development of lung cancer and nicotine dependence [8,60].

### 2.4. Heart

Multiple CYP enzymes relevant to drug metabolism or bioactivation are present in cardiac tissue in low or moderate amounts, including CYPs 1A1, 1B1, 2C8, 2C9, 2D6, 2E1, and 3A4 [31]. Interestingly, higher CYP mRNA expression (e.g., 2D6, 2C) in right ventricular tissue vs. left ventricular tissue, which is indicative of increased drug inactivation at this site, has led some researchers to suggest that differential expression may contribute to therapeutic failure in pharmacological treatment of right ventricular hypertrophy [45]. However, the most prevalent CYP isoform in the heart is CYP2J2, an enzyme involved in metabolizing the anticancer drug, doxorubicin [31]. One common adverse effect of doxorubicin is cardiotoxicity, an effect that may be mitigated in cases of CYP2J2 over-production in cardiomyocytes [61]. Further, cardiac CYPs 1A1 and 2J2 have been shown to be induced in mice following treatment with cocaine and Bap [62].

### 2.5. Blood

CYP mRNA and protein, including that of CYPs 3A4, 2A6, 2E1, 1A1, and 1B1, have been observed in human monocytic and lymphocytic cells [13,21,22,50,51], with 2A6 being the most abundantly expressed isoform in monocyte-derived macrophages [21]. Monocytes are part of the mononuclear phagocyte system, a family of myeloid lineage that also includes macrophages and dendritic cells [63]. These blood cells are critical in host defenses against pathogens, as well as in maintaining tissue homeostasis [64]. Blood monocytes may mature into macrophages under inflammatory conditions and migrate to tissues, where they can synthesize and secrete inflammatory mediators [63]. CYP2A6 induction has been observed in monocytes derived from the plasma of smokers [50], and 2A6-mediated metabolism of nicotine is associated with increased oxidative stress and DNA damage in monocytic cells [13,50]. Similarly, cigarette smoke condensate (CSC) induced CYPs 1A1 and 1B1 in in vitro monocyte studies [51]. In addition, alcohol-inducible CYP2E1, which is known to metabolize alcohol in the liver, was also found to be expressed and induced by alcohol in monocytes/macrophages, leading to oxidative stress [21].

### 2.6. Brain

Total CYP protein content in the brain is substantially lower compared to the liver and is estimated at 0.5%–2% of hepatic CYP content [65]. Although the contribution to systemic drug metabolism may be minimal, brain CYP activity and variation may have a significant impact on local metabolism and the therapeutic efficacy of centrally acting drugs, including antidepressants, antipsychotics, drugs of abuse, and carcinogens [66]. CYP enzymes are differentially expressed in some regions of the brain and in neurons and glial cells. The highest CYP content is found in the brain stem and cerebellum, and the lowest in the striatum and hippocampus [67]. The proposed functions of cerebral CYPs vary by cell type and location.

#### 2.6.1. Neurons

Drug metabolizing CYPs 1A1, 2B6, 2E1, and 3A4 are found primarily in neurons [65], while CYP2D6 is expressed in pyramidal neurons in addition to glial cells [68]. CYP3A4 is involved in psychotropic drug metabolism, including antiepileptic metabolism [65,68]. Considering that alcohol dehydrogenase (ADH) is not expressed in the brain, while CYP2E1 is constitutively expressed in various brain regions, it is probable that CYP2E1 is the brain’s major alcohol metabolizing enzyme [69].

#### 2.6.2. Monocytes and Glial Cells

CYPs are abundant in astrocytes at the blood–brain barrier (BBB), aiding in the regulation of xenobiotic influx into the CNS, blood flow modulation, and signaling during inflammatory conditions [70]. Notably, CYP1B1 is expressed on cerebral micro-vessels and astrocytes at the BBB interface [71,72], and in conjunction with membrane transporters, may aid in regulating xenobiotic passage into and out of the brain. CYP2D6 is expressed in neurons and glial cells [68]. CYP2D6 is involved in opioid metabolism and that of many antidepressants, antipsychotics, and detoxification of pesticides [66,73]. Further, in vitro studies have shown that CYPs 3A4, 2A6, 1A1, and 2E1 are expressed in human monocyte-derived-macrophages as well as in astrocytic cell lines [13,49,50,51,52]. CYPs 1A1 and 2A6 account for the majority of CYP content in SVGA astrocytes, while CYP2A6 is the predominant isoform in U937 macrophages [13,21]. Monocytes are known to enter the brain from the periphery and differentiate into perivascular macrophages and microglia [74], making their presence in the CNS an important target for CYP activity.

Relatively low levels of brain CYP content may have neurotoxic ramifications. For example, low cerebral 3A4 may make brain tissue more sensitive to pharmaceutical inhibition of psychotropic drug metabolism, as illustrated by ritonavir-mediated inhibition of carbamazepine and consequent ataxia [75]. Moreover, a possible explanation for nicotine-mediated induction of 1A1 and 2A6 observed in astrocytes, but not hepatocytes, may be that hepatic 1A1 and 2A6 are already expressed at maximal levels [13]. Indeed, brain CYPs seem to be particularly sensitive to xenobiotic-mediated induction. Increased expression of CYPs 2E1, 2B6, and 2D6 has been observed in the brain tissue of smokers and alcoholics [14,69,76,77,78], but changes in hepatic 2B6 and 2D6 are undetectable [78,79]. Our in vitro study showed that CYP2E1 is induced by alcohol exposure in both astrocytes and monocytes via oxidative stress-mediated protein kinase C/c-Jun N-terminal kinase/specificity protein1 (PKC/JNK/SP1) pathways, which eventually causes cellular toxicity. Although evidence suggests CYP2E1-mediated mechanisms of cellular toxicity, including neurotoxicity and contribution to neurological diseases (HAND, PD), it is possible that that cellular CYP content in the CNS is insufficient to meditate these effects. Therefore, it is worth considering the possibility of additional sources of CYP enzymes, which may be transferred to brain cells, when evaluating xenobiotic-induced toxicities and therapeutic efficacy in brain cells.

## 3. Circulating CYP Enzymes and Their Role in Cell–Cell Communication

### 3.1. EVs and Their Origin

EVs are small, membrane-bound vehicles of intercellular communication that carry various types of cellular information throughout the body. The role of EVs in cell–cell interactions is rather complicated; thus, EVs may influence the pathophysiology of recipient cells in either positive or negative ways [80]. EVs comprise a heterogenous group and are therefore classified into three major categories based on their biogenesis pathway: exosomes, micro-vesicles, and apoptotic bodies [29]. Exosomes (<200 nm), which originate from endosomal compartments, are secreted from cells when micro-vesicular bodies (MVBs) fuse with the plasma membrane, whereas micro-vesicles (50–1000 nm) are routinely shed from cell membranes, and apoptotic bodies (50–5000 nm) are released during membrane blebbing upon programed cell death [29]. Due to overlaps in size, composition, and marker proteins, exosome characterization has proved to be challenging for researchers [30]. Therefore, we will exclusively refer to “EVs”, even when published data refers to “exosomes”.

### 3.2. Role of Circulatory CYPs in Drug Metabolism and in Cell–Cell Communication

Produced by and secreted from cells into extracellular plasma, EVs transmit genetic material, proteins, and other biological cargos that reflects the function of the organ from which they originate [24,81]. Once EVs exit the cell by exocytosis, they travel to distant cells via biological fluids such as plasma, cerebrospinal fluid, and urine, where they fuse with recipient cells [81]. The cargo is then released and is free to exert its effects on target cells [81,82]. This transmission of EVs throughout the body provides a means of communication between cells—offering a new source of biomarkers, as well as a potential tool in characterizing variability in drug exposure and therapeutic intervention [24,82].

Furthermore, studies have revealed that EVs carry a multitude of drug metabolizing enzymes, including members of the CYP enzyme group [23,83,84] (Table 2). However, the presence and amount of CYP enzymes is likely to vary greatly depending on the EV source, whether the EVs are isolated from plasma or a specific cell line, as well as the physiological condition of the cells from which they originate [85].

Our group recently detected CYPs 1B1, 2A6, 2E1, and 3A4 mRNA in plasma-derived EVs from healthy subjects, with 2E1 displaying > 500-fold higher expression than the other CYPs identified. We also detected CYPs 1A1, 1B1, 2A6, 2E1, and 3A4 at the protein level [23]. In our studies, plasma EVs were isolated using 0.22 μm filtration, followed by different methods including single and double isolations with a commercial kit [23,26], in addition to the ultracentrifugation method [92]. Further, absolute spectra revealed a higher level of CYPs in plasma-derived EVs versus plasma alone, which indicates specific packaging of CYPs within circulating plasma EVs. Importantly, activity assays confirmed the enzymatic activity of EV CYP2E1 and 3A4. Interestingly, our finding indicated a higher level of CYP2E1 in plasma EVs than in liver cells/EVs, which is the powerhouse of CYP enzymes. The plasma EV CYP2E1 level was also higher than alcohol-induced CYP2E1 in monocytes. Together, these findings suggest that EV packaging is carefully regulated. A study performed by Rowland et al. further strengthened our findings, demonstrating the presence of peptides and mRNA of CYPs, 1A2, 2B6, 2C8, 2C9, 2C19, 2D6, 2E1, 2 J2, 3A4 and 3A5, UGT 1A1, 1A3, 1A4, 1A6, 1A9, 2B4, 2B7, 2B10 and 2B15, and NADPH-cytochrome CYP reductase in plasma-derived exosomes [86]. As EVs act as intercellular messengers, this differential packaging has a crucial impact on the pathophysiology of the recipient cells, and an abundance of CYP enzymes in EVs suggests their necessity at points across the body.

#### 3.2.1. Alcohol, Acetaminophen and EV CYP2E1

Circulating CYPs likely play a role in extrahepatic metabolism. Once secreted from the liver, EVs can deliver CYP enzymes to distant sites, where they then affect the target cells by influencing the metabolism of pharmaceutical drugs, drugs of abuse, and other xenobiotics [23]. CYP2E1 is mainly found in the liver, where it is known to be a major metabolizer of alcohol and acetaminophen (APAP) [12]. Importantly, CYP2E1-mediated metabolism produces reactive oxygen species (ROS) responsible for oxidative liver damage and cellular toxicity [12]. While alcohol and APAP misuse are significant contributors to liver damage, they also affect extrahepatic tissues [93]. Moreover, alcohol induces EV release from hepatocytes in association with ROS [94]. Our study has also shown that alcohol-exposure to mice induces the level of EV CYP2E1 in the plasma [87]. We have further demonstrated that when plasma EVs containing increased levels of CYP2E1 are exposed to hepatic and monocytic cells, they exacerbate alcohol- and APAP-induced toxicity [87]. We have also shown that the toxicity is mainly caused by CYP2E1, as a CYP2E1-selective inhibitor significantly reduced EV-exacerbated toxicity by both alcohol and APAP. Together, the results suggest that EVs containing CYP2E1 can cause both intra- and intercellular communication. Thus, CYP2E1’s presence extrahepatically and its role in cell–cell communication suggests that alcohol and APAP are also being metabolized at different locations throughout the body. It is likely that plasma EVs transmit CYP2E1 from the liver to targets throughout the body, e.g., the brain, where 2E1 can then participate in xenobiotic metabolism and bioactivation of toxic metabolites. EVs carrying CYP2E1 to distant cells may help explain alcohol-induced cellular injury occurring outside the liver.

#### 3.2.2. Tobacco Smoking and EV CYP2A6, 1B1, 1A1

CYP2A6, CYP1B1, and CYP1A1, all of which play a vital role in the metabolism and bioactivation of tobacco/cigarette smoke constituents, were also detected in EVs [23]. Although CYP2A6 and 1B1 are mainly expressed in the respiratory system, they can also be found in liver cells. Thus, EV CYP2A6 and 1B1 may originate from either organ. Both produce toxic metabolites in association with their roles in metabolizing nicotine and PAHs, respectively [10]. Our recent studies suggest that EVs may also play a defensive role, specifically in protecting against smoking-induced HIV-1 pathogenesis [92,95]. CYP-mediated elevations in oxidative stress that accompany tobacco smoking, also promote HIV-1 replication [13,14,50,51]. Additionally, cigarette smoking is associated with EV release in smokers and in various cell types in vitro [96]. Our study revealed that EVs from CSC-treated cells were found to alter their antioxidant capacity and packaging—showing a protective effect against toxicity and viral replication in the early stages of HIV-1 replication [92].

#### 3.2.3. Drug Metabolism and EV CYP3A4

Importantly, we also detected metabolically active CYP3A4 enzyme in plasma exosomes [23]. Being the major drug-metabolizing CYP enzyme, the presence of CYP3A4 in EVs has clinical significance in terms of therapeutics. During drug development, the focus is traditionally on hepatic drug metabolism; however, failing to account for circulating CYPs may result in unintended drug–drug interactions or toxicity. Furthermore, EV CYP3A4 can be used as a biological marker, specifically in examining the metabolism of pharmacological or illicit drugs. Rowland et al. demonstrated a strong relationship between EV CYP3A4 and drug clearance in patients, which suggests that EVs can be a potential tool for identifying variability in drug exposure [86]. The study also found that EV CYP3A4 exhibits comparable kinetics to microsomes taken from liver samples [86]. The circulation of EVs in bodily fluids allows for greater accessibility in terms of isolating these biomarkers. Sometimes called “liquid biopsy,” this form of sample collection does not require the use of invasive techniques such as tissue biopsy or liver resection. Rather, to assess the expression of CYP3A4 mRNAs, they can simply be isolated from a blood sample. These findings suggest that EV CYPs may provide a new and easier way to explore variability in pharmaceutical drug metabolism and exposure.

#### 3.2.4. Biological and Clinical Significance of CYP Packaging/Circulation in Plasma EVs

Although it is recognized that EVs can envelope functionally active CYP enzymes, the specific mechanistic pathway of this differential packaging is still under investigation. Circulating EVs with metabolically active CYP enzymes may have a considerable impact on neighboring and distant cells and tissue systems. For example, CYP enzymes carried within EVs might influence the metabolism of endogenous and xenobiotic compounds. EVs could be way of removing unwanted CYP enzymes from the cells. Further investigations are warranted to fully appreciate the impact that these modified EVs may have in the body [83]. EVs derived from patient hepatocytes can be utilized as a non-invasive tool to characterize variability in drug response—one way in which EVs may be used as potential biomarkers [83]. Further, EVs are already under investigation to be used as drug delivery systems, designed to contain specific content for transport to different cell types [97]. Thus, EVs might be a useful tool in combating xenobiotic-induced toxicity by controlled alteration of their contents.

## 4. Potential Applications of EVs Containing CYP Enzymes

### 4.1. Circulating CYP Enzymes as Biological Markers of Drug-Induced Toxicity

The current gold standard biomarkers for hepatic injury are based on the measurements of hepatic enzymes levels, including ALT, AST, etc., in plasma or serum. However, the ALT levels do not always correlate with various stages of liver disease due to its relatively short half-life [98]. Therefore, specific components in circulating EVs may have great utility as non-invasive biomarkers for diagnosis and during treatment of hepatic injury. For example, alcohol use increases CYP2E1 expression in plasma EVs [88] and there is a correlation between increased CYP2E1 level and alcohol-induced liver injury [99]. Furthermore, alcohol exposure increases EV release into the circulation [88], making circulatory EVs a potential source of biomarkers in the setting of drug-induced liver injury [84,100].

Recent studies show that much like alcohol’s effect on the liver, alcohol can also alter EV cargo [93]. Furthermore, EVs derived from alcohol-treated cells have been shown to exacerbate disease progression through the delivery of altered cellular material to target cells. We previously observed that EVs collected from mouse and human plasma aggravated alcohol and APAP-induced toxicity [87]. In a similar study, Cho et al. demonstrated that CYP2E1-rich EVs from alcohol-exposed rats and patients induced hepatic cell death [88]. These findings further highlight the potential value of CYP2E1-containing EVs as noninvasive, diagnostic biomarkers in alcoholism and microsomal stress [101].

Similarly, smoking induces CYP1A1 [58], CYP1B1 [102], and the activity of these enzymes can exacerbate smoking-related toxicity by providing additional oxidative stress [58]. Since these CYPs are present in EVs [23,86], EVs can serve as markers to diagnose smoking-induced tissue toxicity. Similarly, a strong relationship between EV-derived CYP3A4 and drug clearance in patients [86] suggests that EV CYP3A4 can be used as a biological marker, specifically in examining the metabolism of pharmacological or illicit drugs.

### 4.2. Use of EV CYPs in Synthetic Biology

**Figure 2:**
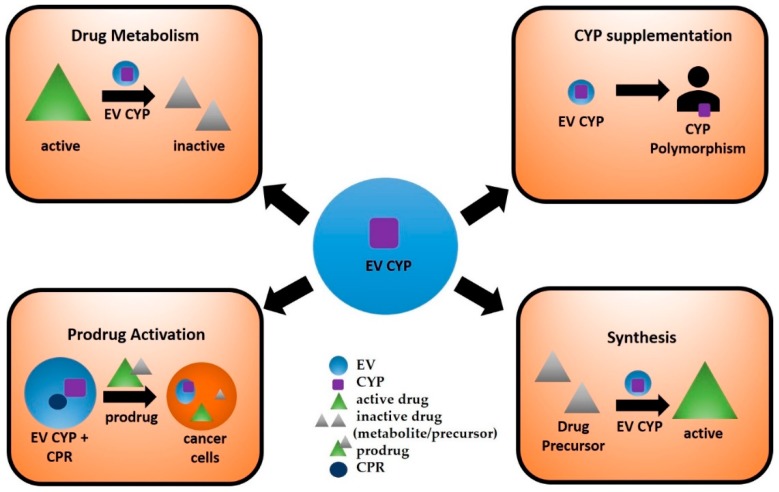
Potential applications of extracellular vesicles containing CYP enzymes include drug metabolism, prodrug activation, supplementation of CYP to subjects with genetic polymorphisms, and industrial synthesis of biomolecules.

CYPs can catalyze the specific addition of oxygen atoms to chemical scaffolds, which would be very challenging and expensive by traditional methods. Several CYPs and engineered variants are now used to synthesize and produce various compounds on a larger scale and for diverse purposes, including drug discovery and development [1]. For instance, artemisinic acid, a precursor for the *Artemisia annua*-derived antimalarial drug, artemisinin, has been synthesized using an engineered form of the plant’s CYP71AV1 enzyme [103]. Furthermore, engineered CYPs have utility in statin synthesis. Compactin is a naturally-occurring HMG-CoA reductase inhibitor originally isolated from *Penicillium citrinium* by Endo et al., 1976 [104]. Using an engineered version of *Amycolatopsis orientalis*-derived CYP105AS1 in *Penicillium chrysogenum* fungi, researchers are now able to synthesize pravastatin from compactin [105]. Moreover, as the proteins packaged in EVs are stable and protected from degradation [106], engineered CYPs can be loaded in EVs (Figure 2), a step that would improve their stability and subsequent activity in the production of various therapeutic molecules.

### 4.3. Targeted Delivery of EV CYPs for Prodrug Activation

The ability of EVs to package and transport a variety of biological cargos has prompted investigators to examine the possibility of loading EVs with specific therapeutic content [107]. Several methods of EV loading have been developed, including electroporation, transfection, and incubation, among others [108]. Utilizing one of these methods, it is conceivable that EVs may be loaded with CYP enzyme, along with CPR. The EV-loaded CYP and prodrugs can be directly administered to the site of disease. For example, in the case of solid cancer, EV CYP can activate anticancer prodrugs at the disease site, reducing toxicity in healthy cells caused by anticancer drugs. Engineering such a delivery system could enhance the efficacy and bioavailability of certain prodrugs, cancer treatments (including brain cancer), and neurological disease therapies.

Previously, gene-directed enzyme prodrug therapy (GDEPT), which utilizes gene transfer of CYP enzyme and cytochrome CPR within a viral vector, has been proposed as a novel way to increase therapeutic efficacy and decrease systemic side effects of anticancer prodrugs, e.g., cyclophosphamide (CPA) and ifosfamide (IFA) [1]. The purpose of CYP-based GDEPT is to facilitate local CPA/IFA bioactivation by expressing CYP enzymes directly within tumor cells [109]. While initial trials of CYP-based GDEPT systems have demonstrated safety and enhanced chemosensitivity to tumors, no GDEPT products are currently on the market [110,111]. As EVs are already under investigation as potential delivery systems [97], it is possible that EVs loaded with CYP and CPR could replace the viral vector in CYP-based GDEPT systems (Figure 2). The approach of loading EVs with CYP and CPR would be safe and economical due to the biological origin of EVs. Moreover, several therapeutics fail to achieve optimal concentrations in the CNS due to their inability to cross the BBB. In such instances, EVs can be engineered to target CNS cells and deliver their contents. For example, in delivering a prodrug along with its activating CYP enzyme to microglial cells, EVs could be conjugated with anti-TEME119 antibody [112], which is specific to microglia, to target and deliver EV cargo to these cells. Further, bacterial CYP enzymes have been expressed and engineered to activate prodrugs [1]. Loading of these CYPs in EVs targeted to a particular tissue can increase their stability and further promote their prodrug-converting activity in target sites.

### 4.4. Delivery of EV CYPs to Supplement Naturally Inactive CYPs 

EV-loaded CYPs can also be administered to subjects with loss of function polymorphisms for particular CYP enzymes (Figure 2). Genetic polymorphisms of drug-metabolizing enzymes can result in either decreased, increased, or complete lack of activity of an enzyme, leading to disease susceptibility [113,114] or variability in drug response [115,116,117]. CYP2D6, which is known to metabolize approximately 20% of drugs, is the most polymorphic CYP enzyme in many ethnic populations and varies from 1–50% [118]. Several CYP2D6 variants cause very low to no activity with several drugs [119]. Similarly, CYP3A5 contributes significantly to drug metabolism in humans and is not expressed in 90% of Caucasians [120]. Thus, certain drugs, e.g., tacrolimus and sirolimus, that are metabolized by CYP3A5, tend to accumulate and cause toxicity in most Caucasians [120]. Thus, administrating EVs loaded with CYP3A5 to Caucasians could be helpful in metabolizing 3A5 substrates and decreasing their respective toxicities.

### 4.5. Current Challenges Associated with Using EVs as Therapeutics

Although EVs have advantageous properties over synthetic delivery systems in terms of their biological source and ability to deliver functional cargo, clinical translation of EVs as diagnostic or prognostic markers of pathological states remains a challenge due to various reasons. One reason might be the lack of uniformity in isolation, characterization and analysis methods of EVs. This can lead to variations in EV counts and phenotypes between different laboratories, making data analysis and clinical translation difficult. Furthermore, the half-life of exosomes in athymic nude mice was reported to be 30 min, and clearance was estimated to be 6 h after intravenous injections [121]. However, due to compartmental changes as EVs travel throughout the human body, it is difficult to estimate the half-life of EVs in blood. Moreover, EVs from different cells and EVs with different sizes possess different biodistribution profiles. In addition to all these concerns, most studies regarding the physiological or pathological effects of EVs have been done in cell culture models. However, cells under in vivo conditions are under a constant steady-state exposure to EVs. Therefore, the extent to which controlled EV exposures under in vitro conditions corresponds to the in vivo environment remains unclear.

The purification and detection of EVs is improving with the help of technological advancements. Moreover, International Society for Extracellular Vesicles (ISEV) attempts to provide guidelines to isolate and characterize EVs in order to improve reproducibility and to avoid ambiguity in the identification of EVs [122,123]. EVs can be engineered in order to increase their circulation time and improve their delivery to target tissues. For example, EVs can be coated with polyethylene glycol, which is known to increase the half-life of nanoparticles [124]. Increased expression of CD47 on the EV surface can also improve the circulation time of EVs by opposing the actions of phosphatidylserine, which promotes the initiation of phagocytosis and subsequent removal from the circulation by macrophages [125,126]. Therefore, exploring EV circulation kinetics, targeting, internalization, and cell–cell trafficking routes will be useful in engineering EVs for therapeutic purposes.

## 5. Conclusions

Considering the profound contribution of CYP enzymes in mediating xenobiotic metabolism and bioactivation of toxicants, the presence of CYP enzymes in EVs and their biological significance cannot be ignored. As EVs circulate throughout the body via biological fluids and participate in cellular communication, they may be clinically useful as biomarkers for drug-induced toxicity, synthesis of drug/metabolite synthesis, and targeted prodrug activation. Thus, further investigating the roles of circulating CYPs in extrahepatic cells would help generate novel treatment options for neurological diseases, cancer, and more.

## Figures and Tables

**Table 1:** Select xenobiotic metabolizing cytochrome P450 enzymes expressed in human extrahepatic tissues.

Tissues/Organs	CYPs Detected	References
Small intestine	3A4/5^+/++/+++^, 1A1^+/++^, 1B1^+^, 2C9^+/++/+++^, 2C19^+/++/+++^, 2D6^+^, 2E1^+^	[32,33,34]
Kidney	2B6^+/++/+++^, 3A5^+/++/+++^	[6,35,36,37]
Lungs	1A1^+/++/+++^,1A2^+/++/+++^, 1B1^+/++^, 2A6^+/++/+++^, 2B6^+^, 2C^+/++^, 2D6^+/++/+++^, 2E1^+/++/+++^3A4/5^+/++/+++^	[38,39,40,41,42,43,44]
Heart	1A1^+^, 1A2^+/++^, 1B1^+^, 2C8^+/++^, 2C9^+/++^, 2J2^+/++^, 2B6/7^+^, 2D6^+^, 2E1^+/++^, 3A4^++^	[31,45,46,47,48]
Blood (monocytes and lymphocytes)	1A1^+/++^, 1B1^+/++^, 2A6^+/++/+++^, 2B6^+/++^, 2D6^+/++^, 2E1^+/++^, and 3A4/5^+/++/+++^	[13,49,50,51,52]
Brain	1A1^+/++^, 1A2^+/++^, 1B1^+/++^, 2A6^+/++/+++^, 2B6^++^, 2C8^+^, 2D6^+/++/+++^, 2E1^+/++/+++^, 3A4/5^+/++^	[13,14,15,53]

Key: + mRNA, ++ protein, +++ activity.

**Table 2:** Select xenobiotic metabolizing cytochrome P450 enzymes expressed in EVs.

Tissue/Organ/Fluid	Human/Animal	CYPs Detected	Isolation Method/References
Plasma	Human	1A1^++^, 1A2^+/++^, 1B1^+/++^, 2B6^+/++^, 2A6^+/++^, 2C8^+/++^, 2C9^+/++^, 2C19^+/++^, 2D6^+/++^, 2E1^+/++/+++^, 2 J2^+/++^, 3A4/5^+/++/+++^	Total Exosome Isolation Kit (from plasma) [23], exoEasy Kit (membrane affinity spin column) [86]
	Mouse	2E1^++^, 3A4^++^	Total Exosome Isolation Kit (from plasma) [87] (unpublished data)
	Rat	1A1^++^, 1A2^++^, 2E1^++^, 4A^++^, 4B^++^	Ultracentrifugation [88]
Hepatocytes	Human	2E1^+/++^	Total Exosome Isolation Kit (from cell culture media) [23]
	Rat	2A1^++^, 2A2^++^, 4A2^++^, 2B3^++^, 2C11^++^, 2D1^++/+++^, 2D3^++^, 2D18^++^, 2D10^++^, 2D26^++^	ExoQuick Kit [89] Ultracentrifugation [84,90,91]
Monocytes/Macrophages	Human	2E1^+/++/+++^, 1A1^+/++^, 2A6^+/++^	Total Exosome Isolation Kit (from cell culture media [23,92]

Key: + mRNA, ++ protein, +++ activit.

## Abbreviations

EVs	Extracellular vesicles
CYP	Cytochrome P450
CPR	CYP reductase
APAP	Acetaminophen
ROS	Reactive oxygen species
CSF	Cerebral spinal fluid
Bap	Benzo(a)pyrene
PAH	Polycyclic aromatic hydrocarbon
NNK	Nicotine-derived nitrosamine ketone
CSC	Cigarette smoke condensate
ADH	Alcohol dehydrogenase
BBB	Blood-brain barrier
CNS	Central nervous system
HAND	HIV-associated neurocognitive disorders
PD	Parkinson’s disease
MVBs	Micro-vesicular bodies
ALT	Alanine aminotransferase
AST	Aspartate aminotransferase
GDEPT	Gene-directed enzyme prodrug therapy
CPA	Cyclophosphamide
IFA	Ifosfamide
